# Extra Supplement.—The Nursing Mirror

**Published:** 1888-10-27

**Authors:** 


The Nursing Mirror.

All Contributions for this Supplement should be addressed "The Nursing Mirror," care of The Editor, 38, Old Jewry, E.C.

### En Passant.

FLORENCE NIGHTINGALE.—The smallest item of news concerning Miss Nightingale is always eagerly received by every nurse throughout the land, therefore we state that she has written an interesting letter to the Public Health Society of India, expressing sympathy with its aims, and strongly urging it to show persistence in the work of sanitary reform.

FIFTY POUNDS WANTED.—Anyone interested in the question of district nursing should make a point of visiting the Nurses' Home, Ipswich, where Miss Pye has instituted an excellent system of teaching the poor to nurse their own sick. The plan is to lend all such sick-room necessaries as may be needed, and to arrange for a trained nurse to visit each case as often as may be required. This excellent scheme is capable of wide extension, and Miss Pye is in immediate need of an extra £50 (say 400 half-crowns) to establish it on a sound basis. Most people could spare half-a-crown for such a work as this, and we hope that the £50 will soon find its way to Miss Pye, at the Nurses' Home, Ipswich.

THE BRITISH NURSES' ASSOCIATION.—We are glad to hear that this association will admit reporters to its meetings in future, for we shall now be able to keep our readers *au courant* with its affairs, as they have often asked us to do. At the last meeting it was announced that the Association had £300 in hand, but not more than eleven hundred and fifty members, including doctors, matrons, sisters, and nurses, of whom there must be at least forty thousand in this country who are eligible. With a daughter of the Queen for president, and the President of the College of Surgeons and all the Bartholomew nurses to back them, we confess we had looked for greater results.

EMIGRATION.—Through a misprint in our last number it was stated that nurses in the Sydney Home received *five* instead of *ten* shillings a-week when engaged in the home. The following is a copy of the agreement a nurse signs before sailing: "I, ——, hereby agree as follows: (1) To leave London for Sydney, New South Wales, by the —— steamship, sailing on the ——. (2) On my arrival, to enter the service of the Sydney Home and Training School for Nurses, Phillip Street, Sydney, for the period of one year, to be computed from the date of reporting myself at the above-named Home, at a salary of 10*s*. per week while engaged at the Home; 20*s*. per week while engaged on active service away from the Home in ordinary cases; and 30*s*. per week while engaged on active service away from the Home in infectious cases. (3) Board, lodging, washing, and uniform to be provided for me. (4) And I further agree to conduct myself in a satisfactory manner during the aforesaid period of one year, and to obey all reasonable orders and directions of the managers, and others placed in authority over me for the time being, of the above-named Sydney Home and Training School for Nurses. As witness my hand, &c," Lady Samuel hopes to persuade the Orient Company to grant nurses reduced passages after December; at present this company's ships are all full. The outfit necessary is merely what any woman would require for a long voyage. There are no arrangements for sending the nurses home should they desire it; that they would be left to undertake entirely by themselves. A regular wave of emigration seems moving our nurses at present, and we can only hope they will all find the Colonies the El Dorados they seem to imagine them. Sisters Cotton and Currie, of the London Hospital, sail for Adelaide next month.

OBSTRUCTING A MONTHLY NURSE.—Among several curious applications to the magistrate at Dalston Police-court lately was one by a gentleman who said his landlady threw every possible obstacle in the way of a monthly nurse who was attending his wife, and that, as a consequence, he was afraid his wife's life would be imperilled. Mr. Horace Smith replied that he did not see what he could do in the matter, but he would send a policeman to caution the landlady.

A MEMORIAL TO MRS. BROMHEAD.—An admirable portrait of Mrs. Bromhead (late lady-superintendent of the Lincoln Nursing Institution) has just been placed in the Bromhead Memorial Building, recently erected by public subscription on the Nettleham Road, Lincoln. The portrait is given by the present and former nurses of the Lincoln Institution, in memory of their venerated lady-superintendent, and bears on its frame the following incription: "This Portrait of Mrs. Anne Fector Bromhead, Founder and Lady-Superintendent of the Institution for Nurses, Lincoln, was presented by the Nurses of the Institution, in Reverent and Grateful Memory, and as a Token of Affectionate Respect for their Present Lady-Superintendent, her Daughter, Miss Henrietta Bromhead. Lincoln, October, 1888." It is an excellent likeness, and the work is of a high order of merit. Mr. Lockhart Bogle, of Bushey, Herts, pupil of Professor Herkomer, A.R.A, was the artist entrusted with the execution of the portrait, which was painted solely from photographs. The Bromhead Memorial Building, opened in April last, was erected to "commemorate Mrs. Bromhead's benevolent work in creating, developing, and administering 'The Institution for Nurses,' which has been established in Lincoln for the last twenty years; and it is universally acknowledged that this Institution has proved a very great help and blessing to many thousand persons of every rank and class, not only in the City and County of Lincoln, but throughout England."

THE EMPRESS FREDERICK AS NURSE.—"His Majesty had a large staff of attendants, but with the exception of the orderly, who was on duty in the sick-room at night, there was no trained nurse among them. The Emperor had four personal attendants, two valets, and two jägers, besides his chief personal attendant Wetterling, who had been his soldier-servant almost since the time he joined the army thirty-seven years previously. Wetterling gave a general superintendence, administering the medicines, and arranging about the food under my directions. The Empress herself took an active part in the nursing, showing a practical knowledge of the art equal to that of any trained nurse. Her Majesty had several times given excellent proof of her skill and gentleness in dealing with the sick and wounded in the military hospitals during the bloody campaigns of 1866 and 1870; and now, when her own gallant soldier-husband was laid low, she managed the details of the sick-room in a way that filled us all with admiration. Hitherto the assistance which the Empress had given us, though of the greatest value, was more of a moral than a practical kind. Often, indeed, when we doctors were losing heart, her Majesty would cheer us up and stimulate us to fresh efforts by her courage and example; but now her struggles were really heroic. Often have I seen her wipe away her tears in the Emperor's ante-room, and then go in to him with a smiling face, bringing, as it were, a stream of sunshine into the chamber of suffering, and chasing the look of pain and weariness from the poor patient's countenance. Now her Majesty took a more active part in the work, and showed herself full of devices for ministering to the Emperor's comfort."—From *Frederick the Noble.*

### Facts for Nurses Concerning the General Structure of the Human Body.

No apology is necessary nowadays for attempting to give a popular account of Physiology, and nowhere can such an attempt be more suitable than in a paper largely written for nurses.

Physiology has been well-named the "Doctrine of Life," since it deals with those organs in the body without which life and action would be impossible, and the absence of which would reduce us to inanimate statues. What strikes us most in any mammalian animal, whether human or not, is the capacity for movement: he can stand up, sit, or lie down at will; and we naturally ask ourselves, Why have this class of animals, commonly called vertebrated, this power, which is denied to the invertebrated animals, such as reptiles, etc.? The difference depends entirely upon the presence or absence of a bony framework and of a vertebral column; and these consist of a number of bones, each distinct from the other, but united by muscles and tendons which answer the double purpose of holding them together, and of allowing movement to the various limbs by acting as levers. Another and quite as important use of the skeleton is to protect the internal organs within—such as the heart, lungs, and abdominal viscera.

The next point in the skeleton which strikes us, is the wonderful manner in which the bones are constructed to exactly fulfil the purpose for which they are intended. In the body we find a great variety of bones as regards both size, shape, thickness, and strength, and they are commonly classified as flat, long, short, or irregular. Of flat bones, we have two such different types as the pelvic and lachrymal bones, the latter being one of the thinnest in the body; and were these two placed side by side it would be difficult to believe that the same term, "flat," ought to be applied to each. Of long bones the femur and the humerus are the best types, whilst of short bones those of the hand and foot, and of irregular bones those of the wrist and ankle and of the vertebral column are good examples. As regards strength, the bone best calculated to bear weight and with the greatest amount of resistance is the femur, which has a rectangular head and strong shaft, which is not made straight: but, on the contrary, is slightly curved, which gives it elasticity and spring, just as a bow is curved. Thus the femur has great "weight-bearing strength," but its power of movement is more circumscribed than that of the humerus, which has no weight to bear, but requires great "pulling-strength," and variety of movement — for we often have to swing our arms round, but only acrobats use their legs in a similar manner, and most people are content with merely backward and forward movements. Again, the skull is intended to protect the brain beneath, and therefore its bones are made thin, very strong, and at the same time light: if they were heavy, in proportion to their strength, we should, have trouble in maintaining an upright position, and if we ever ventured to advance our head should run great risk of falling forwards—in fact, we should be top-heavy, and our head and its due balance would be a constant anxiety. With our hands, composed of many bones, we have great flexibility, and power of doing delicate work, which would be impossible were our hands one large bone. These few instances will suffice to show how well bones are adapted for the part each one is intended to play. Naturally, all these elaborate bones require centres of nourishment and government, and the former are found in the heart, lungs, and digestive system—the latter in the brain and spinal cord. The nourishment is carried from these centres to the most distant parts of the body, by means of an intricate system of blood-vessels, varying in size from a large quill to the finest thread; and so close is this network, that it is impossible to insert a needle anywhere without transfixing one at least of them, and causing bleeding. In the same way, communication with the brain and spinal cord and other parts of the body is carried on by means of the nerves, which cover the body with a complete network.

The heart is a muscular bag, usually described as the "size of the closed fist of the owner," and capable of rhythmical contraction. It is divided longitudinally into two distinct halves, which do not communicate; and laterally it is again divided, but these divisions communicate by valves, which have the power of closing and preventing the blood from escaping. In the left side is the pure arterial blood, which by the contractile power of the heart is forced into the arteries, and dispersed by means of these and the capillaries all over the body, regenerating the wasted tissue in its course, supplying muscle-forming constituents to muscle, nerve-forming constituents to nerve, and to bone what will nourish it. And in place of what it thus loses, gathers up waste products, which are returned to it through other vessels, very similar to the arteries, but called veins, and poured into the right side of the heart—not, however, to remain there, but forced by the contractions of the heart into another set of vessels which convey it to the lungs, where it undergoes that mysterious process of oxygenization which repairs much of the loss; after which it is returned to the left heart, and once more goes on its round.

But what is this blood that is so necessary to the welfare of the body? To the naked eye it appears as a red fluid, and if a drop be freshly drawn from the finger and placed on a saucer it is quite still. Examine it under a microscope, however, and it will appear to be a reddish-yellow fluid, composed of innumerable spherical bodies, which, at least for some time after the blood has been drawn, will appear rolling about, and the pale-coloured ones will change their shape. The use of the red bodies, or corpuscles, is to attract oxygen, with which the blood comes into close contact in the lungs, and to hold a large quantity of this gas in solution, whilst the use of the white corpuscles is to nourish the tissues.

How does the blood contained in the vessels nourish the surrounding tissues? If we covered a piece of land just below the surface with a network of water-pipes it would not make the soil more fertile, and why should it be otherwise with the pipes containing blood? It is merely an illustration that fluids can penetrate a moist membrane. The arteries and veins have not this power, for their coats are too thick; but the arteries break up into ever smaller capillaries, with very thin walls, and thus the blood can traverse them, supply the wants of the surrounding tissues, be re-absorbed into other vessels, and enter the stream of the venous blood. Other organs that assist in the renewal of, the blood by restoring the various materials lost to the tissues are the digestive organs. After all, man cannot live only on air, nor would his blood be of much service if it was only kept supplied with oxygen. A properly regulated diet will contain a due proportion of all the elements which go to form the body; and this food, during the process of mastication, is so acted upon by the saliva, and later by juices in the stomach and intestines, that it is changed into a soluble form which can penetrate into the blood. The liver is the largest gland in the body, and it supplies a fluid, the bile, which plays a very important part in the digestive system. The process of absorption in the intestines is very complicated. The food, which leaves the stomach in a partly digested form, enters the small intestines, where the bile and pancreatic fluid are poured on it, and make it perfectly soluble. The small intestines, which are about 20 feet long, have their walls studded with minute glands, called Villi, which are so constructed to absorb the food easily; and in order to increase to the utmost the absorptive surface, it is arranged in folds which increase it at least fourfold. In close contiguity with these glands is a blood-vessel, into which the food enters readily. Of the excretory organs, the skin, and kidneys, we cannot now do more than merely make a bare reference, but some future time we shall hope to go fully into the working of these organs.

### Everybody's Opinion.

[*Correspondence on all subjects is invited. No communications can be entertained if the name and address of the correspondent is not given, or unless one side of the paper only be written on.*]

A CONVALESCENT HOME FOR NURSES.—Is it not high time that we, the overworked nurses, should be provided with a Convalescent Home of our own, where we could enjoy each other's society and benefit by each other's experience? Would you kindly give us a suggestion how to proceed in obtaining one? and oblige,—NORWICH.

THE BRITISH NURSES' ASSOCIATION.—If "Dear Nurse" will pay a five-shilling subscription to the Midwives' Institute and Trained Nurses' Club, 15, Buckingham Street, Strand, she will get the use of a club-room, medical papers, good library, and splendid lectures in return for her money. —A MEMBER.

THE PRIVATE NURSE.—Whilst "A Nurse's Position" is being discussed, may I ask, is it the rule or the exception for the private nurse to take her meals in the kitchen? I think if one and all made a firm stand against it we should not much longer be so insulted. I am sure all nurses agree with me when I say we do not wish to become acquainted with the family details which it is the pleasure of domestics to retail at those times.—A PRIVATE NURSE WHO HAS FOUND IT THE RULE.

A MELBOURNE HOSPITAL.—I see in your issue of October 13th an account of the Alfred Hospital at Melbourne. The following is my personal experience of that admirable institution. I had been in the colony only two months when I was unlucky enough to catch typhoid, and acting on the advice of the doctor who attended me in my lodgings, I went to the Alfred Hospital. I think I owe my life to this move, for it would have been quite impossible for me to have obtained in my rooms the constant and untiring care and attention I received at that hospital. I was at first placed in the general paying ward; but my case becoming evidently critical, I was moved, and on coming to myself some weeks afterwards found myself comfortably lodged in a large and airy private room, with a hospital nurse sitting at my bedside. It would be impossible for me to say too much in praise of the kindness and consideration of this nurse; and, indeed, of all whose duty it was at any time to take charge of me. I am bound to say, however, that I shall always be an advocate of lady nurses, for the blessing of the tender, and at the same time cheerful, sympathy of educated and intelligent nurses is too real not to be distinctly acknowledged. The house physician was always most attentive, paying me visits regularly twice a-day, and sometimes oftener. When I was sufficiently recovered, I was carried on a lounge on to the balcony of the hospital on fine mornings (in Australia there are such things), and I shall never forget the delightful feeling of exhiliration I experienced on these occasions. That my case was serious, the following will show: A pupil nurse said to me, 'You have been such a good case, sir. Yours has been the most complicated case I have yet seen, and I have learned a great deal from it.' Truly there are two ways of looking at things! I must say that I think there is too much typhoid in Melbourne, and that the authorities should remember that prevention is better than cure.—A PATIENT FOR TWELVE WEEKS.

### NURSES AND FEES.

"They like fees, no doubt ought to like them; yet, if they are brave and well educated, the entire object of their live is not fees. They, on the whole, desire to cure the sick; and if they are good doctors or nurses, and the choice were fairly put to them, would rather cure their patient and lose their fee than kill him and get it. Their work is first, their fee second. But there is a vast class who are ill-educated, cowardly, and more or less stupid, and with these the fee is first and the work second. If your work is first and your fee second, work is your master, and the Lord of work, who is God. But if your fee is first and your work second, fee is your master, and the lord of fees, who is 'the least erected fiend that fell.'"—*Ruskin.*

### Appointments.

[It is requested that successful candidates will send a copy of their applications and testimonials, with date of election, to THE EDITOR, The Lodge, Porchester Square, W.]

BEDFORD INFIRMARY.—Miss F. M. Newberry has been appointed matron of this infirmary, out of fifty-one applicants. Miss Newberry trained at the Royal Infirmary, Newcastle-on-Tyne, where she took an excellent certificate, and then went to Bradford for a year as charge nurse. Afterwards Miss Newberry went as sister to the Hospital for Women and Children, Waterloo Road, where she finally became matron. For the last two years Miss Newberry has acted as matron at the Yeatman Hospital, Sherborne. We congratulate Miss Newberry on her steady rise in her profession.

PENDLEBURY CHILDREN'S HOSPITAL.—Miss Dennis, who trained at the London, has been appointed night superintendent of this hospital.

GATESHEAD CHILDREN'S HOSPITAL.—Miss Harper, who trained at Guy's, has been appointed matron of this Jubilee hospital.

### Lectures.

ON October 30th, and following Tuesdays, at Taunton, a course of six lectures on "Water and its History," by Mr. V. Perronet Sells, M.A., F.C.S.

The following Gilchrist People's Lectures may interest our country readers: A course of twelve lectures on "Physiology" will be delivered this term by Mr. G. E. A. Parkyn, M.A., at Lancaster, Blackburn, and Halifax. A course of three lectures on "Air and Food in Relation to Life," will be concluded by Mr. Parkyn, at Bolton, on October 30th; at Chorley, November 2nd; and delivered at Crewe, November 6th, 13th, and 20th. Admission, 1*d.* per lecture.

At the Midwives' Institute and Trained Nurses' Club, Dr. McNaughton Jones will continue his excellent series of lectures for the next two months.

### Vacancies.

The following vacancies are announced:—

### Nurses.

Cumberland Infirmary.

Central London Throat and Ear Hospital; £20.

Victoria Home, Bournemouth.

Cancer Hospital, Brompton; £25.

St. Saviour's Infirmary; £20.

Cheltenham Nursing Institution.

Staffordshire Infirmary.

Canterbury Nurses' Institute.

Arbroath, N.B., Infirmary; £20.

Blackheath Institute for Trained Nurses; several.

Nursing Institute, St. Heliers, Jersey; several.

Workhouse Infirmary Nursing Association, 6. Adam Street, Strand, W.C.: several.

Yeovil District Hospital; £14.

West Bromwich District Hospital, two; £22.

Firs Home, Bournemouth; £20.

Bradford Infirmary; two; £35.

County of Cornwall Nurses' Home.

Nursing Institution, 96, 97, 98*a*, Wimpole Street, London, W., Mr. Wilson has several vacancies for thoroughly-trained Nurses.

Swansea and South Wales Nursing Institution.

Nottingham and Notts Nursing Institution; several.

Nursing Institution, 3, Delamere Crescent, Paddington; several.

Poplar Hospital for Accidents.

Newlands, Ryde, I.W.

### District Nurses.

Goldings. Newcastle, Staffordshire. Dublin.

### Probationers.

Arbroath, N.B., Infirmary; £10.

Peterborough Infirmary.

Birmingham Children's Hospital.

Workhouse Infirmary Nursing Association. 6, Adam Street, Strand, W.C.; several.

### Matron.

Borough of Colchester Urban Sanitary Authority Hospital for Infectious Diseases.

### Notes and Queries.

### Queries.

(72) *Blind Asylum.*—Can anyone inform me of an asylum where a man totally blind from an accident will be taken free of charge?—*Douglas.*

(73) *Hip Disease.*—Can any nurse give me her experience with these sad cases? At what stages was treatment begun, how long was it carried on, and how often was it successful?—*A.M.*

(75) *Addenbrooke's.*—Can any of your correspondents give me any information about Addenbrooke's, Cambridge? How many nurses are there—special, and nurse probationers? Are any ever kept on with salary after one year's work as probationers? Are there any exams. to pass, and is the work as hard as in the London hospitals?—*Madge.*

### Answers.

*Books on Midwifery.*—Nurse E. should get Cullingworth's Manual J. and A. Churchill. Price 1s. 6d.

*Volunteer Nurses.*—*Volunteer* will find Surgeon-Major Evatt's paper in a book called *Common Accidents.* Chatto and Windus, 1*s.*

